# 가상현실 기반 해부학 교육에 관한 국내 간호대학생의 경험: 질적 내용분석

**DOI:** 10.7586/jkbns.25.002

**Published:** 2025-02-25

**Authors:** Dallong Han, Gyeong Ju An

**Affiliations:** Department of Nursing, Cheongju University, Cheongju, Korea; 청주대학교 간호학과

**Keywords:** Virtual reality, Qualitative research, Students, nursing, Anatomy, 가상현실, 질적 연구, 간호대학생, 해부

## Abstract

**Purpose:**

This study explored the experiences of nursing students in Korea regarding virtual reality (VR)-based anatomy education.

**Methods:**

A qualitative study was conducted with 103 first-year nursing students who had participated in immersive VR-based anatomy education. Nursing students wore head mounted displays and controllers, and performed VR-based practice for 10 minutes per student. The submitted materials were analyzed using the qualitative content analysis method developed by Elo and Kyngäs.

**Results:**

The collected materials were classified into three themes, six categories, and 14 subcategories. Three themes are emerged: (1) amazing experiential learning about the human body, (2) challenges in VR-based anatomy education, and (3) expandability of VR-based anatomy education. These results showed that nursing students who participated in VR-based anatomy education experienced “amazing” experiential learning about the human body in this new approach, with the vividness and knowledge consolidation of VR. However, nursing students experienced challenges in VR-based anatomy education related to the unfamiliarity and limitations of VR. Furthermore, they experienced the expandability of VR-based anatomy education by strengthening learning motivation and new approach to nursing education.

**Conclusion:**

This study will help in planning VR-based education in human anatomy by exploring the experiences of nursing students. It is suggested that VR-based education in human anatomy be expanded and implemented as part of regular anatomy practice within the curriculum rather than a one-time educational intervention.

## 서론

### 1. 연구의 필요성

의료정보기술의 끊임없는 발전과 더불어 간호교육 분야에도 가상현실(virtual reality, VR)을 활용하는 개인별 교육을 적용하려는 시도가 점진적으로 증가되기 시작하였다. 간호학 실습 교육과정에 가상 시뮬레이션 실습 프로그램이 이용되고 있으며, 그 효과도 다양한 연구들을 통해 검증되고 있다. 국내 간호대학생을 대상으로 가상 시뮬레이션 실습교육에 관한 연구들은 대부분 3-4학년을 대상으로 가상 시뮬레이션(virtual simulation)을 이용하여 임상 상황과 비슷한 가상 시뮬레이션 안에서 시나리오에 따라 환자와 상호작용하는 방식으로 분만여성 대상자[1], 성인간호 대상자[2,3], 정신간호 대상자[4,5] 시뮬레이션 프로그램의 효과를 양적 연구와 질적 연구를 통해 확인하였다. 가상 시뮬레이션 프로그램의 도입은 학생들이 안전한 환경에서 간호사의 역할을 반복함으로써[1,5] 문제중심으로 사고하는 지식의 확장 효과가 있고[2,3] 임상수행능력과 비판적 사고가 증가한다[4]는 연구보고가 있었다. 즉, 이러한 가상 시뮬레이션은 이론적인 간호지식을 통합할 수 있도록 도와주며, 실제 환자를 대상으로 하지 않기 때문에 안전한 교육방법으로 간주된다. 특히 Z세대라 불리는 디지털시대의 대학생들에게 적합한 교육방법이 될 수 있으나[6], 실제 상황 같은 느낌이 부족하다는 단점이 제기되었다[7]. 이에 따라 좀 더 실제와 같은 느낌을 경험할 수 있도록 헤드마운트디스플레이(head-mounted display, HMD)인 VR 헤드셋을 착용하는 몰입형 가상현실(immersive VR) 프로그램을 간호교육에 적용하는 연구들이 점차 활발해지고 있으며. 특히 해부학 교육에 몰입형 VR 프로그램을 적용하는 연구가 활발해지기 시작하였다.

간호학 교육과정 중 인체의 구조와 기능에 대한 이해는 전문직 의료인이 되기 위해서 필수적으로 알아야 할 기본적인 기초간호과학 지식이다[8]. 하지만 학생들은 ‘인체의 구조와 기능’이나 ‘인체해부학’ 교과목을 암기해야 할 의학용어가 많아 힘든 교과목으로 인식하여, 시험을 위한 단순 암기를 목적으로 피상적인 학습을 하는 경우가 많다[9]. 따라서 인체의 구조에 대한 이해를 높이기 위해 카데바를 이용한 해부학 교육이 병행되고 있지만, 카데바 실습 비용과 시간이 많이 요구되고 의과대학이 없는 간호학과에서는 접근하기 어려워 모든 간호대학생이 경험하기에는 기회가 제한적이다[10]. 따라서 이러한 어려움을 해결하기 위한 노력으로 국내 간호대학에서 몰입형 VR을 이용한 해부학 교육프로그램의 적용이 확대되고 있다.

특히 코로나바이러스감염증-19 (coronavirus disease 2019) 팬데믹 이후 대면 카데바실습이 어려워진 의과대학에서 몰입형 VR기반 인체해부학 교육이 증가된 이후 국내외 의과대학생[11-13]을 비롯하여 치위생학과 학생[14,15], 작업치료학과 학생[16] 등 의료보건계열의 여러 학과에서 VR기반 해부학 교육을 시행한 효과에 대해 연구결과를 보고하였다. 선행연구에서 의과대학생 일부에서는 몰입형 VR기반 해부학 교육에 대한 만족도가 낮고 이러한 프로그램이 카데바 실습을 대체할 수 없다고 보고한 반면[11] VR기반 인체해부학 교육에 만족도가 높고[14] 긍정적인 해부학 교육방법으로 보고하기도 하여[12-16] 상반된 결과가 나타나기도 하였다.

간호대학생을 대상으로 수행된 몰입형 VR기반 해부학 교육에 관한 연구로는 국외 간호대학생들에게 VR기반 해부학 교육을 적용 후 해부학적 지식과 만족감이 유의하게 증진되었다는 보고가 있었다[17,18]. 국내에서는 VR기반 해부학 교육에 관한 양적인 연구가 주로 이루어졌는데, VR기반 해부학 교육 적용 후 간호대학생의 학습실재감, 기술수용성, 해부학 지식이 유의하게 증가하였다고 보고한 연구[19]와 VR기반 해부학 교육의 사용성에 영향을 미치는 요인에 대해 조사한 연구[20]가 보고된 바 있다. 그러나 몰입형 VR기반 해부학 교육에 참여한 국내 간호대학생의 관점에서 교육 대상자인 간호대학생이 어떠한 경험을 했는지에 관해 탐색한 연구는 찾기 어려웠다.

카데바 실습이 점차 어려워지고 보건의료 수요 증가로 간호대학생이 증원되는 상황에서[20] 앞으로 VR기반 해부학 교육프로그램의 활용이 확대될 것으로 예상할 수 있다. 따라서 이러한 교육방법에 대한 간호대학생의 구체적인 경험에 대한 이해는 기본적으로 고려해야 할 부분이다. 즉, 간호대학생에게 효과적인 해부학 교육 프로그램 개발을 위한 기초자료로서 몰입형 VR기반 해부학 교육에 참여한 학생들의 주관적이고 생생한 실습 경험에 대한 면밀한 파악이 요구된다고 볼 수 있다. 따라서 본 연구는 선행 양적 연구로는 확인하기 어려운 몰입형 VR기반 해부학 교육에 참여한 국내 간호대학생의 주관적인 경험을 파악하기 위해 시행되었으며 질적 내용분석 연구방법을 활용하여 이를 탐색하고자 한다.

### 2. 연구 목적

본 연구의 목적은 몰입형 가상현실을 이용한 해부학 교육에 참여한 간호대학생의 주관적인 경험을 탐색하기 위한 것이다. 이를 위한 본 연구 질문은 ‘간호대학생의 몰입형 VR기반 해부학 교육 참여경험은 어떠한가’이다.

## 연구 방법

### 1. 연구 설계

본 연구는 일 대학 간호학과 학생들을 대상으로 인체 구조와 기능 교과목에서 몰입형 VR기반 해부학 교육에 자율적으로 참여하게 한 후 자유롭게 작성한 성찰일지를 대상으로 질적 내용분석(qualitative content analysis)을 수행한 질적 연구이다.

### 2. 연구 대상

본 연구의 대상자는 목적표집(purposive sampling)을 이용하여 청주대학교 간호학과의 1학년으로 1학기에 ‘인체 구조와 기능I’ 교과목을 수강한 후, 2학기에 ‘인체 구조와 기능Ⅱ’ 교과목을 수강하면서 본 연구 참여에 동의한 간호대학생 103명이었다. 질적 내용분석 연구에서 연구대상자 수는 정량적으로 정해져 있지 않기 때문에[21] 전체 103개의 성찰일지를 읽으면서 연구자가 알고자 하는 내용을 분석하였다.

### 3. 자료 수집

**Figure 1.**
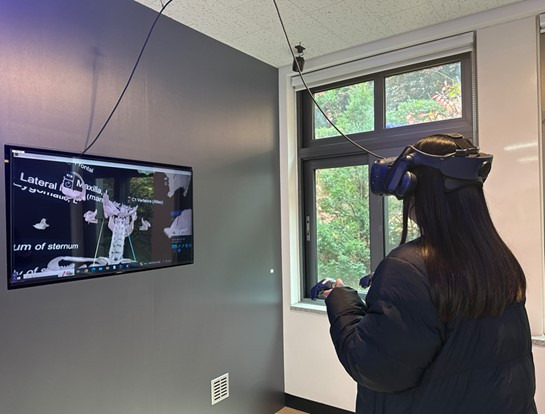
A nursing student directly participating in virtual reality-based anatomy education.

본 연구를 위해 간호학과 1학년 학생 108명에게 연구 목적과 방법을 설명하고 그중 연구 참여에 자발적으로 동의하는 103명의 동의서를 받았다. 자료 수집 기간은 2024년 11월 25일∼12월 6일까지였으며 ‘인체 구조와 기능Ⅱ’ 교과목을 수강하는 간호대학생들을 교내에 있는 VR 실습실에서 몰입형 VR기반 해부학 교육프로그램으로 해부교육을 경험하게 하였다. 미리 예약한 시간에 6∼7명 학생들이 연구원의 조작법 오리엔테이션을 들은 후, 3군데 VR 스테이션에서 자율적으로 VR기반 인체해부학 교육프로그램을 활용하여 관찰하기를 원하는 계통이나 장기를 자유롭게 개인당 10분간 헤드셋을 쓰고 실습을 하였다(Figure 1). 본 연구에서 사용된 VR 프로그램은 3D Organon VR anatomy (3D Organon software, Medis media, Queensland, Australia)로 인체를 15개 계통과 일만개가 넘는 부위로 구성하여 해부학 형태를 영어 설명과 함께 관찰할 수 있게 되어 있으며[16], 헤드셋과 컨트롤러(HTC VIVE Pro Fullkit, HTC Corporation, Taoyuan, Taiwan)를 착용하여 관찰하였다.

성찰일지의 질문은 3개의 개방형 질문으로 ‘본인이 진행한 실습은 무엇입니까?’, ‘본인의 활동 소감은 어떻습니까?’, ‘VR기반 해부학 교육에서 바라는 점은 무엇입니까?’였고, 학생들이 실습 종료 직후 해당 내용을 형식 없이 자유롭게 기술하여 연구원에게 제출하게 하였다. 각 학생들이 작성한 성찰일지는 A4용지 2/3∼1쪽 분량이었으며 총 103장이 자료 분석에 활용되었다.

### 4. 자료 분석

질적 내용분석은 대상자의 서술형 자료를 분석하여 코딩을 하고 분류하는 과정을 통해 자료의 범주화로 잠재적인 의미를 분석할 수 있는 질적 연구 유형 중 하나로[22], 방대한 질적 자료를 쉽게 분석할 수 있는 방법이기 때문에 학생들의 성찰일지를 분석하기 위해 본 연구에 적합한 방법이다.

본 연구에서는 질적 내용분석의 여러 연구방법 중 Elo와 Kyngäs [23]의 3단계 귀납적 내용분석 과정을 이용하였다. 첫 번째 준비 단계에서 분석 단위(unit of analysis)를 찾기 위해 수집된 자료를 반복하여 읽었으며 메모를 하면서 귀납적 접근을 통해 분석 단위인 의미있는 단위(meaning unit)를 확인하였다. 이 단계에서 “하나씩 떼어봄”, “여러 각도에서 관찰”, “신기함” 등 의미있는 분석 단위들을 확인하였다. 두 번째 조직 단계에서 분석 단위를 조직적으로 배열함으로써 동일한 의미를 가지고 있는 분석 단위를 확인하고 그룹으로 묶어서 범주화시켰다[23]. 세 번째 보고 단계에서는 하위범주를 다시 그 의미와 특성의 유사점과 차이점에 따라 통합한 후 더 높은 차원의 범주로 추상화시켜 주제(themes)를 이끌어내는 것이다. 질적 내용분석이란 자료라는 매개물에 담긴 내용에 대한 타당한 추론을 이끌어 내는 것이므로[24] 이 과정을 연구자들이 심도깊게 숙고하고 논의하는 과정을 거쳐 진행하였다.

### 5. 연구의 타당성 검토

본 연구는 Lincoln과 Guba [25]가 제시한 신뢰성(credibility), 전이가능성(transferability), 의존성(dependability), 확인가능성(confirmability)의 평가기준을 고려하여 타당성을 확보하였다. 첫째, 신뢰성을 충족하기 위해 성찰일지는 참여자들이 실습 경험 직후 자유로운 분위기에서 작성하도록 하였으며, 자료분석 과정에서 연구자들은 참여자들이 성찰일지에 작성한 표현과 추출한 의미 단위들이 일치하는지 상호점검하며 참여자의 경험 그대로를 연구결과 도출에 반영하고자 노력하였다. 또한, 도출된 연구결과를 연구에 참여한 3인에게 보여주어 개인의 경험과 일치하는지를 확인하였다. 둘째, 전이가능성을 충족하기 위해 간호학과에서 인체해부학 교육을 주로 받는 학년인 1학년 학생, 남녀 성별을 모두 포함한 대상으로 목적 표집하였으며, 연구가 이루어진 환경, 연구에 활용된 VR기기 및 소프트웨어 등의 연구 맥락을 상세히 제시하였다. 셋째, 의존성 확보를 위해 자료수집 및 분석 시 Elo와 Kyngäs [23]가 제시한 방법에 따라 자료수집 및 분석과정을 거쳤으며 이를 자세히 기술하였다. 질적 내용분석 논문을 국내외 학술지에 다수 게재한 경험이 있는 두 연구자가 각각 추출한 의미 단위를 모아 범주화하고 주제를 찾아내는 과정에서 다수의 논의와 합의를 거쳐 결과를 도출하는 지속적 상호점검 과정을 거쳤다. 넷째, 확인가능성을 확보하기 위해 자료분석 과정에서 연구자가 연구주제에 관한 가정이나 선입견을 갖지 않도록 지속적으로 성찰하며 가치 중립적인 태도를 유지하였으며, 연구자의 선입견이나 주장을 나타내는 표현은 없었는지 확인하였다.

### 6. 윤리적 고려

본 연구를 위해 연구자들이 소속된 청주대학교의 기관생명윤리위원회 심의와 승인을 받았다(IRB No.1041107-202410-HR-039-01). 연구 대상자들의 윤리적 보호를 위해 연구 시작 전에 제공된 연구목적과 방법에 대한 설명문을 읽고 연구 참여는 언제든지 자발적으로 참여를 중단할 수 있고 이로 인해 어떠한 불이익도 받지 않음을 설명하였다. 또한, 모든 자료를 연구 목적으로만 사용되고 비밀 보장이 유지될 것임을 설명한 후 자발적으로 연구 참여에 동의하는 참여자에게 서면동의를 받은 후 진행하였다. 참여자들이 학생이므로 성적평정이 끝난 후 자료 분석을 시작하였으며 연구 종료 후 수집된 성찰일지는 개인 저장장치에 자료를 보관하여 연구자만 접근 가능하도록 하였다.

## 연구 결과

### 1. 대상자의 일반적 특성

본 연구 대상자인 간호학과 1학년 103명의 연령은 18∼23세 범위로 평균 20.8세였으며, 남학생 14명(13.6%), 여학생 89명(86.4%)이었다. 대상자 중에서 헤드셋을 이용한 VR 게임 등을 이용해 본 적이 있는 학생은 64명(62.1%)이었고, 39명(37.9%)은 VR 프로그램을 직접 사용해본 적이 없었다. 연구 대상자들이 관찰한 인체 계통은 심혈관계가 27.9%로 제일 빈도가 높았고 근골격계 18.4%, 신경계 12.2%, 소화계 11.8%, 비뇨생식계 10.7%, 호흡계 6.1%, 피부계 5.2%, 감각계 4.7%, 림프계 1.9%, 내분비계 1.1%의 순서로 VR기반 해부학 교육을 한 것으로 나타났다.

### 2. 질적 내용분석 결과

연구대상자들의 VR기반 해부학 교육 참여 후 작성한 성찰일지에 관한 질적 내용분석 결과 59개의 의미 단위, 14개 하위범주, 6개 범주, 3개 주제가 도출되었고, 3개 주제는 ‘인체에 대한 놀라운 체험 학습’, ‘VR기반 해부학 교육에의 도전’, ‘VR기반 해부학 교육의 확장성’으로 도출되었다(Table 1).

#### 1) 주제 1: 인체에 대한 놀라운 체험 학습

본 연구에 참여한 참여자들은 강력한 실재감과 몰입감으로 대변되는 ‘VR 구현의 생생함’을 체험하였다. 또한 자기주도적이고 능동적으로 인체의 삼차원적(three-dimensional, 3D) 구조를 정확히 이해하여 ‘VR 교육을 통한 지식의 정착’을 경험하였다. 즉, 참여자들은 헤드셋을 통해서 VR 스크린을 보면서 실제 인체를 만지는 듯한 실재감과 인체 내부에 들어가 있는 듯한 몰입감을 통해 놀라워하였고, 자기가 목적한 계통의 장기를 자기주도적으로 컨트롤러를 움직이며 자기 스스로 움직이며 학습함으로써 지식이 견고해지는 경험을 하여 놀라운 체험 학습을 하였다.

##### (1) VR 구현의 생생함

참여자들은 헤드셋을 착용하고 VR 스크린을 통해 실제 눈 앞에 있는 살아있는 인체를 만지는 듯한 강력한 실재감과 인체를 세세하게 탐구하는 몰입감을 경험하였다. 즉, 참여자들은 VR의 특성인 사용자의 시선 움직임에 따라 시야가 실제와 비슷하게 보여지므로 눈 앞에 있는 듯한 실재감과 시각적 생생함을 통해 살아있는 인체를 보는 듯한 놀라운 경험을 하였으며, 주위의 시선이 차단되고 VR 스크린에만 시각적 감각이 집중되므로 깊이 빠지는 몰임감을 느끼게 되었다.

*“생각했던 것 보다 수준과 완성도가 높아서 놀랐고, 퀄리티가 좋아 실제 사람의 인체처럼 리얼하여 내 눈 앞에 있는 기분을 느꼈다. 마치 인체 내부에 들어가 있는 듯한 느낌을 경험하였고 무수한 신경과 혈관을 실제로 직접 만지는 듯한 느낌이 들었으며 생동감 있게 실습을 진행할 수 있어서 좋았다.” (참여자 51)*
*“기존 인체의 구조와 기능 강의시간에 사진, 영상으로만 봤던 것들과 달리 VR기술을 활용하여 인체 구조를 3D로 입체적으로 보니 마치 만지는 듯한 몰입감 덕분에 흥미가 돋았다.” (참여자 19)*

##### (2) VR 교육을 통한 지식의 정착

참여자들은 VR의 360도 삼차원 구조를 통해 인체 장기의 내부 구조, 위치, 모양, 크기를 정확히 이해할 수 있었다. 원하는 계통을 주도적으로 선택하여 필요한 부분을 원하는 만큼 탐색할 수 있어 학습효율이 증가하고, 텍스트와 그림 위주의 정적인 학습에서 벗어나 컨트롤러(스틱)를 직접 움직이며 조직을 하나하나 떼어가며 능동적인 관찰을 할 수 있었다. 학생 스스로 손을 움직여야 학습이 이루어지기 때문에 능동적인 학습과정이 형성된 것이며, 이를 통해 장기의 구조가 옆의 장기와 연결하거나 비교하면서 통합적으로 머리에 각인되고, 수업시간에 배웠던 파편화되어 있던 지식들이 비로소 통합된 지식으로 정착되는 경험을 하였다.

*“VR 실습을 하기 직전에 심혈관계를 배웠기 때문에 배운 내용을 바로 확인하고 싶어 심장을 중점적으로 관찰하였다. (중략) 요즘 심혈관계통을 배우며 심장의 구조가 어려웠는데 내부구조에 대해 더욱 깊이 이해할 수 있어 도움이 많이 됐다.” (참여자 3)*
*“기존의 이론 공부만으로는 이해하기 어려웠던 인체의 복잡한 구조를 3D로 직접 관찰하면서 평소에 궁금했던 자기가 스스로 보고 싶은 곳을 주도적으로 학습하여 보다 명확하게 파악할 수 있었습니다. 특히, 장기와 근육, 뼈의 위치와 관계를 입체적으로 이해하게 되어 해부학적 지식을 더욱 확실하게 다질 수 있었습니다. (중략)내가 직접 손쉽게 다양한 각도에서 해부할 수 있어 학습의 효율성이 크게 향상되었습니다.” (참여자 74)*
*“화면에서 인체 구조를 스틱으로 직접 하나씩 떼어가며 본 것이 기억에 남는다. 자세히 못보았던 조직들을 다 뜯어보고 덕분에 오래 머리 속에 남을 것 같다.” (참여자 15)*

#### 2) 주제 2: VR기반 해부학 교육에의 도전

참여자들은 VR기반 해부학 교육의 장점과 더불어 제한점도 기술하였는데, 이는 VR기반 교육을 처음 접함으로 인한 ‘VR 교육의 낯섦’과 ‘VR 구현시 아쉬움’으로 나타났다. 즉, VR기반 해부학 교육을 처음 접해 보면서 작동법이 어려웠고 어지럽거나 울렁거림 같은 VR의 부작용을 경험하면서 VR에 대한 낯설은 느낌을 가지게 되었고, VR 프로그램이 국내제작이 아니어서 영어로만 되어 있는 점, 착용해야 할 VR 헤드셋과 콘트롤러가 무겁거나 불편했던 점, VR 프로그램이 작동 중에 오류가 나서 다시 재시작했다는 등의 VR 구현시 아쉬움을 느끼며 VR기반 해부학 교육에 처음으로 도전해보는 경험을 하였다.

##### (1) VR의 낯섦

참여자들은 VR기반 교육을 처음 접함으로 인해 VR에 대한 어색함이 있었고 아직 VR 컨트롤러조작에 익숙하지 않아 작동법을 익히는 데 다소 시간이 소요되었다. 특히 콘트롤러를 조작하여 아주 작은 부분을 움직이는 것이 힘들고 어려웠다고 표현하여 VR 작동을 미세하게 조절하는 연습이 필요함을 경험하였다. 또한 일부 학생들은 실습 중에 화면의 빠른 움직임 때문에 자주 나타나는 디지털 멀미라고 일컬어지는 약간의 어지러움, 울렁거림 등의 VR기반 게임이나 교육시에 경험할 수 있는 낯선 느낌을 경험하기도 하였다.

*“스틱 조작이 어려워 내가 보고자 하는 곳을 보기까지가 조금 어려웠기 때문에 아쉬운 점도 있었다. 사용법을 익힐 수 있는 시간이 따로 있었으면 좋겠다. 조준하기가 조금 어려워서 조작이 좀 더 편했으면 좋을 거 같다.” (참여자 89)*
*“조금 어지럽긴 했지만 충분히 가치가 있었던 수업이었던 것 같습니다.” (참여자 25)*
*“글로만 배웠을 때에는 크게 와닿지 않았었는데 실제로 VR로 봤을 때는 울렁거리며 비위가 안좋아질 정도로 생생해서 신기했다.” (참여자 63)*

##### (2) VR 구현시 아쉬움

참여자들은 모든 인체구조의 이름과 설명이 영어로만 되어 있어 이해하기가 어려웠다는 외국 소프트웨어에 대한 불만, VR 기기의 무게, 작동상의 잦은 오류 등 하드웨어상의 한계를 경험하였다.

*“3D 인체해부학 실습시 모든 것이 영어여서 약간 불편하였기 때문에 한글로도 하였으면 좋겠다.” (참여자 31)*
*“한글이 안되고 VR기기가 무겁다는 점이 아쉬웠습니다.” (참여자 2)*
*“오류가 생각보다 많이 일어났고 이름이 영어로 되어있어 알아보기 힘들었다.” (참여자 14)*

#### 3) 주제 3: VR기반 해부학 교육의 확장성

참여자들은 VR기반 해부학 실습이 확대 시행되기를 원하였고 더 나아가 간호술기도 VR기반 교육으로 해보고 싶다는 간호학 교육에의 확대 적용을 경험했으며, 카데바 실습을 대체할 수 있는 ‘간호학 교육의 새로운 시도’라고 인식하고 있었다. 뿐만 아니라 자신의 전공 지식 부족을 깨닫고 학습에 정진할 필요성을 느꼈으며, 간호학 전공공부에 대한 흥미와 자신감을 내비치는 등 ‘VR 교육을 통한 학습동기의 강화’를 경험함으로써 VR기반 해부학 교육이 단순히 인체의 구조와 기능 지식을 증진시키는 목적 뿐 아니라 간호학 전공 전반에 대한 교육에의 확장성을 경험하였다.

##### (1) 간호학 교육의 새로운 시도

참여자들은 프로그램이 인체를 잘 구현하고 있고 살아있는 신체를 해부하는 듯한 느낌이 생생해 VR기반 해부학 실습이 카데바 실습을 대체할 수 있는 효과적인 방법이라 생각했으며, 해부학 실습 뿐만 아니라 핵심간호술기와 같은 다른 전공 교과목에도 VR실습이 확대 시행되기를 기대하여 간호학 교육에 VR기반 교육이 확장되기를 바랐다. 즉, 향후 간호학 교육에서도 교내에서 VR을 적용하는 전공실습교육의 시도가 시작되기를 바라며 새로운 시도에 대한 경험을 하였다.

*“진짜 사람을 해부한 거 같이 생생했다. 살아있는 인체를 해부하는 느낌을 받았으며 직접 카데바 실습을 가지 않아도 교내에 있는 VR 실습으로도 충분할 것 같다. 실제 해부 실습을 대체할 수 있는 효과적인 방법이라는 생각이 들었다.” (참여자 22)*
*“매우 만족스럽고 앞으로도 이런 VR 실습을 할 수 있는 기회가 많아지기를 기대합니다.” (참여자 77)*
*“인체가 잘 구현되어 있어서 잘 발전시키면 간호실습에도 효과적일 것 같다. VR실습으로 간호 술기를 연습할 수 있기를 바라며 학생들의 간호 능력 발전에 도움이 될 것이다.” (참여자 96)*

##### (2) VR 교육을 통한 학습동기 강화

참여자들은 VR기반 해부학 실습을 통해 자신이 의학용어와 인체에 대한 지식이 부족함을 깨닫고 향후 전공 공부에 있어서 해부학 지식이 기반이 되어야 하므로 더 열심히 학습해야겠다는 동기를 얻었다. 또한 해부학에 대한 흥미와 자신감이 고취됨으로써 VR 교육을 통해 간호학에 대한 학습 동기가 강화되었다.

*“VR 실습을 하면서 이미 인체의 구조를 이해하고 실습하거나 의학용어를 더 많이 아는 친구를 보면서 자신의 부족함을 깨달았다.” (참여자 7)*
*“인간의 몸이 이렇게나 복잡하고 많은 구조로 되어 있다는게 신기하기도 하였지만, 결국 이걸 다 공부하고 암기해야 자격증을 이수할 수 있겠구나 하는 생각도 들어 학교공부에 더 열심히 임해야겠다는 생각이 들었습니다.” (참여자 48)*
*“그전엔 인간의 내부를 보는 것이 무서웠는데 3D로 보고나서 자신감을 찾을 수 있었다. 3D 인체해부학 실습 덕분에 어렵게 생각했던 인체 구조에 대해 흥미와 학습동기가 또한 더욱 높아졌습니다.” (참여자 81)*

## 논의

본 연구는 Elo와 Kyngäs [23]의 질적 내용분석 연구방법을 이용하여 몰입형 VR기반 해부학 교육에 참여한 간호대학생들의 경험을 탐색하여 향후 간호학 분야에서 인체해부학 교육의 기초자료를 제공하고자 시도되었고, 연구 결과에서 3개 주제와 6개 범주가 도출되었다.

첫 번째 주제인 “인체에 대한 놀라운 체험 학습”은 ‘VR 구현의 생생함’과 ‘VR 교육을 통한 지식의 정착’으로 학생들이 인체를 학습하는 새로운 학습을 경험하였다는 것이다. 즉, 본 연구에서 사용된 몰입형 VR 프로그램의 특성인 생생함으로 마치 인체가 눈 앞에 있는 듯한 감각을 일으킨 것이다. 몰입형 VR기반 해부교육이란 머리에 착용하는 HMD로 컴퓨터가 생성한 가상현실을 시각정보로 전달받아보면서, 시각, 촉각, 청각 등 감각과 상호작용하며 인체 부위를 선택하여 360도 파노라마로 회전, 확대, 축소 등 손으로 조작하며 실제 인간의 시야각과 비슷하게 재현되므로 현실과 구분이 되지 않을 정도로 사실적인 경험을 느낄 수 있게 형성되어있다[6]. 따라서 본 연구의 대상자들은 VR기반 해부학 교육을 처음 참여해보면서 생생함과 삼차원적 입체감을 경험한 것이다. 비록 본 연구대상자 중 가상현실체험형 게임 등 가상현실 콘텐츠를 이전에 경험한 대상자가 전체의 62.1%로 나타나 절반 이상이 VR 체험을 하였지만, 대상자들은 VR기반 해부학 교육 프로그램의 수준과 완성도가 생각보다 높아 놀랐다고 기술하기도 하였다. 이러한 결과는 VR기반 교육을 받아들이는 대상자들의 혁신적인 기술에 대한 수용성과도 관련지을 수 있을 것이다. 선행연구에서 가상 해부절개 테이블(virtual anatomy dissection table) 실습군과 VR기반 해부실습군을 비교한 연구에 따르면 VR기반 해부실습군에서 VR의 높은 실재감 때문에 기술수용성이 더 높게 나타나 새로운 기술에 대한 인식과 사용의도가 높았다고 보고하였다[19]. 또한 간호대학생의 VR기반 해부학 교육 프로그램 사용성에 가장 큰 영향을 미치는 변수는 인지된 혁신성이라고 보고한 연구[20]를 토대로 생각하면, 본 연구에 참여한 간호대학생도 혁신적인 몰입형 VR기반 해부학 교육에 대한 수용도가 높았을 것으로 유추할 수 있다.

본 연구결과에서 VR 교육을 통한 지식이 정착되는 것으로 나타났는데, 대상자들은 삼차원 입체 구조를 관찰함으로써 강의에서 이해가 되지 않았던 부분을 VR기반 교육을 통해 쉽게 이해했다고 하였다. 즉 자유롭게 360도 다양한 각도로 인체 구조를 전후측면으로 관찰함으로써 장기의 정확한 위치, 모양, 크기를 파악할 수 있었다. 이는 해부학을 2차원 교재 그림으로 학습하는 것 보다 삼차원 입체 구조로 학습하는 방법이 해부지식을 유의하게 증가시켰다는 선행연구와 비슷한 맥락으로 볼 수 있다[19]. 그리고 본 연구에서 대상자들이 심장과 혈관을 가장 많이 관찰하였는데 그 이유는 VR 실습을 하기 직전에 이론강의에서 배운 계통이 심혈관계였기 때문에 이론지식을 바로 확인하고 싶었기 때문이라고 진술한 바 있다. 따라서 이론적인 지식과 VR기반 해부학 교육을 효과적으로 연결하기 위해서는 교과목 학습계획시 이론의 계통과 VR의 계통을 매칭하는 VR실습시간을 추가하는 것을 제안한다. 또한, 연구대상자들은 자신이 보고싶은 인체 계통을 선정하고 자기 주도적으로 탐색하면서 학습의 효율성이 크게 향상되었다고 하였다. 일반적인 교내실습은 실습지도자가 옆에서 일일이 관찰하며 가이드하기 때문에 예민하고 자신감이 부족한 학생들은 스트레스를 받을 수 있다[6]. 하지만 VR기반 교육은 기기를 착용한 채 자유롭게 혼자 하기 때문에 자신이 이해하기 어려웠던 부분을 선택하여 확인하고 자기주도적으로 학습할 수 있다. 이러한 자기주도적인 탐색은 수동적인 강의에서 접근할 수 없던 정보들을 자신이 주도적으로 접근할 수 있기 때문에 코드화와 정착이 증진됨으로써 효과적인 학습이 증진된다[26]. 따라서 인체 구조를 자유롭게 자기주도적인 탐색을 통해 관찰함으로써 학습효율성에 영향을 주었다고 유추할 수 있다. 그리고 대상자들은 VR 화면에서 인체구조를 직접 하나씩 떼어가며 본 것이 기억에 남는다고 기술하였다. 즉, 수동적 관찰이 아니라 자기가 직접 조작하는 능동적인 관찰을 통해 학습한 내용이 기억에 남는다는 것이다. 이는 정보처리이론에 따르면 학습자가 단순하게 시각이나 청각을 통해 습득한 감각 기억은 대부분 짧은 시간 동안만 저장하게 되는데, 감각 기억 중 집중을 하여 얻은 정보는 작업 기억(working memory)을 통해 처리되어 장기 기억으로 저장된다[27]. 즉, VR기반 해부학 교육이 단순히 보고 듣는 수동적 관찰이 아니라 학생 스스로가 손을 움직이며(doing) 화면을 조정하여 시각정보를 작업 기억으로 처리하는 능동적 교육이기 때문으로 기억이 오래 갈 수 있을 것으로 생각할 수 있다. 따라서 VR기반 교육은 이미 습득했던 정보를 견고하게 해주는 역할을 하기 때문에[28] 본 연구결과에서 나타난 바와 같이 VR기반 해부학 교육에서 자기주도적으로 능동적인 관찰을 통해 인체 구조를 확인함으로써 지식의 정착을 견고히 해주었다.

이와 같이 VR기반 해부학 교육을 통해 학생들이 경험했다는 지식의 이해와 기억의 정착은 Bloom의 인지 분류체계(Bloom`s cognitive taxonomy)에 따르면 6단계 수준 중에서 1단계 기억하다(remember)와 2단계 이해하다(understand) 수준의 획득에 해당된다[29]. 즉, 간호학 저학년에서 배우는 기초과목인 해부학에서 학습목표를 설정시 인체의 구조를 기억하고 이해하는 것이 중요하므로 인지적 지식 습득 과정에 VR기반 해부학 교육이 도움이 되는 것으로 볼 수 있다.

두 번째 주제 “VR기반 해부학 교육에의 도전”과 관련되어 연구대상자들은 ‘VR의 낯섦’을 표현하였다. 이미 선행연구에서도 의과대학생의 72.7%가 VR기기의 조작이 불편하다는 의견이 나온 결과에 비추어보면[11] VR 헤드셋과 콘트롤러를 처음 사용하는 경우에 학생들이 기기 조작에 어려움을 겪게 된다. 본 연구에서도 실습 시작시에 연구원이 VR 조작법에 관해 오리엔테이션을 했음에도 불구하고 여러 학생들이 처음 경험하기 때문에 낯설어하였다. 이러한 결과는 가상 시뮬레이션 프로그램에 참여한 간호대학생도 조작하는 방법에 익숙하지 않아 어디를 체크해야 될지 몰라서 가상현실에서 혼란을 느꼈다는 보고와 유사하다[1]. 따라서 향후 VR기반 교육을 시작할 때에는 오리엔테이션과 기기 조작에 적응할 시간을 충분히 주는 것이 필요하다고 볼 수 있다.

또한, 어지러움이나 울렁거림을 약간 경험했다는 진술문이 있었는데 의과대학생을 대상으로 수행된 실험연구에서 VR 실험군 학생들이 방향감각이 없어지는 등 시각유도멀미(visually induced motion sickness, VIMS) 같은 부작용이 나타났고[30] 대상자의 9.5%가 어지러움을 호소하기도 하는 등[11] 몰입형 VR기반 교육시 일반적으로 나타날 수 있는 문제이다. 이는 몰입형 VR기반 교육시 입체적인 시각으로 인한 부작용으로 알려져 있다[11]. 본 연구에서는 실습시간을 개인당 10분 정도 시행하였는데 이는 몰입형 VR기반 체험시 VIMS가 나타나는 시간이 대체로 VR 화면에 노출된지 15분 후에 나타난다는 연구결과[31]에 따르면 크게 문제되지 않는 수준이라고 볼 수 있다. 실제로 구토를 하거나 어지럼증으로 실습을 중단한 학생은 없었으므로 본 연구에서 이용한 10분 정도의 VR기반 교육 시간은 적정한 것으로 생각한다. VR 실습시 외부 시각이 차단된 채 혼자 서서 움직이기 때문에 안전의 문제가 있다는 선행연구[28]와 달리 본 연구에서 실습 중 주위에 부딪치거나 하는 안전의 문제를 언급한 학생은 없었다.

그리고 ‘VR 구현시 아쉬움’으로 VR 프로그램의 해부 명칭과 설명이 영어로 되어 있어 이해하기 어려웠다는 아쉬움이 있었다. 이러한 문제는 선행연구[1]에서도 동일하게 나타났는데 시뮬레이션 프로그램이 영어와 의학용어로 구성되어 있어 언어적 장벽이 있어서 간호학생들이 부담스러워하였고 한국어판의 필요성을 제기한 바 있다. 또한 VR 기기의 착용의 불편함과 잦은 작동 오류를 아쉬워하였는데 이러한 결과는 다른 선행연구에서 나타나지 않은 것들이라 이는 일상적이지 않은 상황적인 요인으로 볼 수도 있지만 추후 설문도구를 통해 객관적으로 확인해야 할 필요가 있다고 본다.

세 번째 주제인 ‘VR기반 인체해부학 교육의 확장성’에서 대상자들은 VR기반 해부학 교육이 카데바 실습을 대체할 수 있는 효과적 방법으로 기대한다고 하였다. 이러한 결과는 의대생을 대상으로 한 선행연구에서 VR기반 해부학 교육이 3차원 구조 이해에는 도움이 되지만 카데바 실습을 대체하기는 어려울 것이고 보고한 결과[11]와는 상반된 결과이다. 특히 의대에서는 VR기반 해부학 교육을 카데바 실습 전 단계로 사용하는 것을 제안하였는데, 육안으로 확인하기가 어려운 작은 부위나 내부 구조는 VR로 확인하고 나서 카데바 실습에 참여한다면 더 효과적으로 해부실습을 할 수 있을 것이라고 하였다[11]. 그러나 포르말린 냄새나 카데바의 얼굴이나 몸을 보는 것에 대한 심한 두려움을 갖는 등 카데바 실습이 어려운 학생들이 있으므로[8] 이러한 학생들에게는 VR기반 해부학 교육이 좋은 교육방법이 될 것이라고 생각한다.

또한, 연구대상자들이 VR기반 해부학 교육이 만족스럽고 앞으로 VR기반 해부학 교육 기회가 많아지기를 기대한다고 경험을 기술하였다. 이러한 결과는 의과대학에서 머리뼈 교육시 그림책만 본 그룹 보다 VR 실습그룹과 카데바 실습그룹의 만족도가 높았다는 Chen 등의 연구[32]와 일치한다. 그러나 Chen 등의 연구[32]에서는 VR 실습그룹의 만족감은 높았지만 세 그룹의 성적은 유의한 차이가 없었다고 보고하여 만족감과 해부지식 증가의 연관성은 입증되지 않았다. 다른 연구에서도 의과대학생 대상으로 타블렛을 이용한 그룹과 VR기반 몰입형 해부교육을 한 그룹을 비교한 결과, VR그룹이 성취감이 높았으나 실제 해부지식점수는 유의한 차이가 없는 것으로 나타났다[30]. 본 연구에서는 대상자들의 해부학적 지식을 측정하지 않아 VR기반 해부학 교육의 효과를 객관적으로 확인하지 못하였으므로 추후 연구에서 만족감과 지식을 같이 측정하는 것을 제안한다.

그리고 대상자들은 VR기반 간호술기도 해보고 싶다는 기대가 나타났다. VR의 정의는 HMD와 햅틱 기술을 이용하여 3차원의 가상 환경에서 몰입형 시뮬레이션 경험을 하는 것이다[33]. 현재 국내 간호교육에 적용되고 있는 VR 관련 연구들은 대부분 웹기반 가상 시뮬레이션 프로그램(virtual simulation program)에 관한 연구가 많이 수행되고 있는데 VR이란 용어를 사용하여 혼동되는 경우가 있다. VR은 반드시 헤드셋을 이용하여 VR 몰입의 정도와 사용기기 등이 나타나 있어야 하므로 향후 간호학 분야에서 VR에 대한 정의가 더욱 명확해질 필요가 있다. 그러나 몰입형 VR기반 간호술기 연구는 아직 활발하지 않은 편이며 임상실습을 경험한 고학년 간호대학생을 대상으로 개발된 몰입형 VR기반 간호술기용 교육프로그램에 관한 연구는 분만여성[6], 남성생식질환 대상자[28], 지역사회대상자[34]. 감염관리대상자[35]에게 적용한 연구가 발표된 바 있다. 이러한 연구들에서는 몰입형 VR기반 교육프로그램을 통해 간호대학생의 흥미가 증진되고 지루함이 없어 반복연습으로 위험 상황에서 대상자를 조절하는 간호술기가 능숙해지고 환자안전 문제와 관련된 임상 판단 역량이 강화되어 VR과 같은 상황이 와도 무서워하지 않을 자신감이 증가하는 것으로 보고하였다[1,28]. VR기반 교육에 대한 간호대학생의 교육 요구도를 보고한 연구에서는 안전 요구, 산소화 요구, 배설 요구 등의 다양한 VR 기반 간호교육 콘텐츠를 실습해보기를 원하고 VR기반 교육에 대한 인식도도 높은 것으로 나타나[36,37], 향후 간호술기를 VR기반 교육으로 학습시키기 위한 연구가 활성화되어야 할 것이다.

VR기반 해부학 교육을 통해 대상자들의 학습동기가 강화되었는데, 선행연구에서도 VR기반 해부학 교육을 한 후 학습에 대한 동기 부여와 흥미가 유발되었다고 보고한 바 있다[11]. 그러나 실험연구로 진행된 선행연구[19]에서는 VR기반 해부실험군과 가상해부테이블군의 학습동기에 유의한 차이가 나타나지 않은 것으로 보고하여 상반된 결과가 나타났다. 따라서 학습동기가 증진되는지에 관해서는 향후 양적 연구를 통한 객관적인 평가가 이루어질 필요가 있다. 일부 연구에서는 간호대학생이 VR에 익숙하지 않아서 흥미가 없었다는 연구결과[28]가 있었는데 본 연구 결과에서는 그런 진술문은 나오지 않아 디지털에 익숙한 세대임을 보여주었다. 본 연구에서 이용한 VR기반 해부학 교육은 VR 장비가 구비되어 있는 실습실에서 가능하기 때문에 공간의 제한이 있다. 선행연구에서 모바일 앱을 통해 저렴한 VR 헤드셋을 이용하여 VR기반 교육을 시간과 장소에 제한받지 않고 구현할 수 있다고[6] 하였으나 현실적으로 아직 상용화하기에는 어려움이 있다.

본 연구결과를 바탕으로 고민해봐야 할 부분은 카데바 실습시 학생들이 경험하는 생명 존중의 태도가 강화되는 장점이[8] VR기반 해부학 교육에서 획득될 수 있을 것인가 하는 문제이다. 이는 가상현실에서의 환자가 인간으로 보여지지 않기 때문에 단순하게 기능적으로 접근하기 쉽기 때문이다. 환자의 세계를 환자의 시점으로 보지 않는다면 인간적으로 고려하지 않게 된다. 선행연구에 의하면 몰입형 VR기반 시뮬레이션실습을 수행한 간호학생이 환자에 대한 공감(empathy)을 느꼈다고 보고한 바 있다[28]. 따라서 VR기반 해부학 교육에서도 단순히 지식 습득 뿐만 아니라 인체를 보면서 학생들이 인간에 대한 생명 존중의 태도를 가지도록 교육자가 직접적으로 가이드할 필요가 있다[6].

본 연구에서는 VR기반 해부학 교육을 경험한 대상자의 성찰일지 내용분석을 통해 VR기반 교육의 효과에 대해 양적 연구에서 알 수 없었던 3개의 주제를 통해 구체적인 생각을 확인할 수 있었다. 이와 같은 연구 결과를 토대로 제안한 점은 첫째, VR기반 해부학 교육만이 아니라 카데바 실습을 병행하는 혼합형교육이 학습효과가 더 있을 것으로 생각되나 추후연구를 통해 확인해야 할 것이다. 즉, 가상 시뮬레이션실습이 임상현장실습과 병행될 때 학습효과가 더 높은 것으로 나타난 연구[38]를 토대로 VR기반 해부학 교육에 이어 카데바 실습을 병행하는 교육의 효과를 검증하는 연구를 시행해야 할 필요가 있다. 둘째, 이제는 VR을 간호교육에만 사용할 것이 아니라 자가간호가 필요한 환자교육에도 적용할 수 있도록 VR기반 환자교육프로그램을 개발하여 간호중재에 이용하는 추후연구가 이루어져야 할 것이다.

본 연구의 제한점은 비록 질적 내용분석의 목적은 일반화가 아니라 현상에 대한 이해와 지식 제공이기는 하지만[23], 일 대학의 간호대학생을 대상으로 하였기 때문에 결과 해석에 주의를 기울여야 한다. 또한 VR기반 해부학 교육에 참여하는 학생들이 10분 정도의 일회성 실습에 참여했기 때문에 지속적인 참여 경험은 알기 어렵다. 그리고 자유롭게 개인이 보고자 하는 계통을 선택하게 하였기 때문에 선택한 장기에 따라 느끼는 경험에 차이가 있을 수 있다.

## 결론

본 연구에서는 몰입형 VR기반 해부학 교육에 참여한 학생들의 경험을 탐색함으로써 몇 가지 의미 있는 결과를 얻었다. VR기반 해부학 교육에 참여한 학생들은 VR 체험으로 생생함과 지식의 정착을 통해 인체에 대한 놀라운 체험 학습을 경험하였고, VR의 낯섦과 VR 구현시 아쉬움으로 VR기반 해부학 교육에의 도전을 경험하였다. VR기반 해부학 교육이 끝난 후 간호학 교육의 새로운 시도와 학습동기 강화로 VR기반 해부학 교육의 확장성에 대해 경험하였다.

결론적으로, VR기반 해부학 교육에 관한 간호학 분야의 연구가 부족한 상황에서 본 연구는 기초간호학 교육에 필요한 기초자료를 제공하였다는 의미가 있다. 이러한 VR기반 해부학 교육을 일회성 교육이 아니라 교육과정 내로 흡수하여 구성하여 확대 적용해볼 것을 제언한다.

## Figures and Tables

**Table 1.** Nursing Students’ Experiences with VR-based Education in Human Anatomy

Themes	Categories	Subcategories
Amazing experiential learning about the human body	The vividness of VR implementation	· Strong sense of presence
		· Immersion in exploring the human body
	Knowledge consolidation through VR-based education	· Accurate understanding of anatomy through three-dimensional structures
		· Increased learning efficiency through self-directed exploration
		· Memory enhancement through active observation with hands-on manipulation
Challenges of VR-based anatomy education	The unfamiliarity of VR	· Requiring some time to learn how to operate the VR device
		· The strange feelings of digital motion sickness
	Limitations in VR implementation	· Insufficiency of the software
		· Inconvenience of the hardware
Expandability of VR-based anatomy education	A new approach to nursing education	· Substitution for cadaver dissection
		· Expectation for more VR education
	Enhancement of learning motivation through VR education	· Realization of the importance of medical terminology
		· Awareness of a lack of knowledge about the human body
		· Increased interest and confidence in anatomy

VR = Virtual reality.

## Data Availability

Please contact the corresponding author for data availability.
